# Burnout and Associated Factors Among Dental Students, Interns, and Dental Practitioners in Saudi Arabia: A Cross-Sectional Study

**DOI:** 10.3390/healthcare13131602

**Published:** 2025-07-03

**Authors:** Abdulrahman Mohammed Algethami, Sakeenabi Basha, Roshan Noor Mohamed, Ali Alqarni, Azzah O. Alhazmi, Thani Alsharari, Fahad Saeed Algahtani, Hassan Talat Shawli, Abdullah Amjad Alzamil, Ahmed Sulayyih Alosaimi, Abdulaziz Abdullah Alharbi

**Affiliations:** 1 Faculty of Dentistry, Taif University, Taif 21944, Saudi Arabia; ab.alotaibi7@gmail.com (A.M.A.); alzamil@tudent.org (A.A.A.); kssa949@gmail.com (A.S.A.); 2 Department of Preventive Dentistry, Faculty of Dentistry, Taif University, Taif 21944, Saudi Arabia; roshan@tu.edu.sa (R.N.M.); az.alhazmi@tu.edu.sa (A.O.A.); 3 Department of Oral and Maxillofacial Surgery and Diagnostic Sciences, Faculty of Dentistry, Taif University, Taif 21944, Saudi Arabia; aqarni@tu.edu.sa (A.A.); aa.alharbi@tu.edu.sa (A.A.A.); 4 Department of Restorative Dental Science, Faculty of Dentistry, Taif University, Taif 21944, Saudi Arabia; thani.g@tu.edu.sa (T.A.); f.algahtani@tu.edu.sa (F.S.A.); h.shawli@tu.edu.sa (H.T.S.)

**Keywords:** burnout, dental, students, interns, practitioners

## Abstract

**Background/Objectives**: The present study aims to assess the prevalence of burnout and associated factors among dental students, interns, and dental practitioners in Saudi Arabia. **Methods**: A descriptive cross-sectional study was conducted among dental students, interns, and practitioners at Makkah Province, KSA. The Maslach Burnout Inventory (MBI) scale was used to assess burnout. The difference in mean scores was tested using a *t*-test and analysis of variance (ANOVA). Multivariate logistic regression analysis was carried out for the independent variables and outcome variable of burnout syndrome. **Results**: The total number of participants was 302 (female = 25, male = 277). The mean age of study participants was 30.2 ± 10.1. A total of 66.2% of study participants presented with high emotional exhaustion, 48.7% with high depersonalization, and 38.7% with low personal achievement. Burnout level was 1.78 times (CI = 1.52–3.53, *p* = 0.032) higher among female participants than male participants. Burnout level was 1.53 times (C = 1.31–3.17, *p* = 0.043) higher among student participants compared to practitioners. Burnout level was 2.41 times (CI = 1.72–3.79, *p* = 0.023) higher among participants who worked more than 5 days per week compared to participants who worked ≤ 5 days per week. **Conclusions**: This study’s results showed burnout syndrome was high among dental students compared to interns and dental practitioners. A significant association was seen between increased working hours per week and burnout syndrome. There is a need for a proactive step to highlight the importance of burnout management, especially among dental students.

## 1. Introduction

Burnout syndrome, characterized by emotional exhaustion with feelings of energy depletion, cynicism or feelings of negativism, and reduced professional efficacy [1], has emerged as a significant public health concern within the healthcare sector. The current version of the International Classification of Diseases (ICD-11) defines burnout as an occupational phenomenon, resulting from chronic workplace stress that has not been successfully managed [2]. Recent research reports a concerning prevalence of burnout among dentists, with estimates ranging from 13% globally [2] to 92.8% among dental students in specific regions like Saudi Arabia [3]. Following subgroup meta-analysis, Long et al. [4] suggested the highest level of burnout among European dentists (17%, 95% CI: 5–33), followed by dentists from Asia (12%, 95% CI: 3–25) and the lowest in America (8%, 95% CI: 3–14). Alqahtani et al. [5] reported a prevalence of 46.3% of burnout among Saudi Dental Board residents, highlighting the profession’s potential for burnout across career stages. This trend aligns with broader observations in Arab countries, where Elbarazi et al. [6] documented a range of 20–81% for emotional exhaustion and 9.2–80% for depersonalization among healthcare professionals.

Previous studies have identified various risk factors for burnout among healthcare professionals, including dentists [7,8,9,10,11]. These factors include age, gender, nationality, working hours, service duration, and overall stress levels within the work environment. Importantly, burnout can manifest through symptoms like irritability, anxiety, and depression, potentially leading to negative consequences such as absenteeism, reduced productivity, and increased staff turnover [1,12,13]. Notably, existing research has reported that dentists experience higher burnout rates than other healthcare professionals [2,14,15].

Given the demanding nature of dental education and the potential for negative emotions, understanding the stressors associated with the profession and existing coping mechanisms is crucial [16,17,18]. This study aimed to assess the prevalence of burnout and its associated factors among dental students, interns, and practitioners in Saudi Arabia. By investigating this understudied population, we hope to contribute valuable insights into addressing this critical public health issue in the dental field.

This research is guided by two research questions:

A. What is the prevalence of burnout among dental students, interns, and dental practitioners?
B. How do various sociodemographic and work-related factors influence the prevalence of burnout?

## 2. Materials and Methods

### 2.1. Study Design, Sample Size, and Sampling

A cross-sectional descriptive study was conducted among dental students, interns, and practitioners at Makkah Province, KSA. Ethical clearance was obtained from Taif University with ethical clearance number HAO-02-T-105. The data were collected anonymously and were treated confidentially in accordance with the principles of the Declaration of Helsinki. A pilot study was conducted among 25 subjects (not included in the main study) to pretest the questionnaire and calculate the sample size. With a prevalence of 13%, 80% of the power of the study, and an α error of 5%, a sample size of 273 was needed, which was rounded to 300. A simple random sampling with a lottery method was used to select the final sampling unit. The survey was distributed to 335 participants, and 302 responded (90.1% response rate).

A simple random sampling was performed to select the final sampling unit. A total of 6 colleges were included in the study (3 public and 3 private colleges). From each college, 10–15 students, 5–10 interns, and 10–15 faculty members were selected using the lottery method. The subjects who provided consent to participate in the study were assigned a number, which was randomly selected. A total of 30 healthcare centers (10 healthcare centers each from Taif, Makkah, and Jeddah City) were selected, with 4 to 7 practitioners from each healthcare center. The form was distributed to 335 participants to cover the nonresponse bias, and 302 participants responded to the survey.

### 2.2. Eligibility Criteria

Inclusion criteria: Dental students studying in the 5th and 6th academic year, interns, faculty, and practitioners from the healthcare center who agreed to participate in the study by signing the informed consent form.

Exclusion criteria: Participants suffering from mental illness or psychiatric issues, and those who did not provide signed informed consent.

### 2.3. Questionnaire

The Maslach Burnout Inventory (MBI) scale was used to assess the burnout [1]. The study was conducted from October 2024 to January 2025. The data was collected using a printed questionnaire copy and an online questionnaire. The response rate was 90%. The questionnaire was translated into the Arabic language. Cronbach alpha α was 0.85. The questionnaire included three parts. The first part involved collection of sociodemographic details (age, gender, education, income, Saudi or non-Saudi), the second part involved work-related issues (type of practice, work practice experience, place of work, specialist status as per the Saudi council for Health specialties (SCFHS), duration of work, etc.), and the third part included the MBI scale. The MBI scale includes three dimensions. The responses were rated based on a 7-point Likert scale ranging from never (0) to daily (6).

- Emotional exhaustion (EE; nine items with a sum score range of 0 to 54);
- Depersonalization (DP; five items with a sum score range of 0 to 30);
- Personal accomplishment (PA; eight items with sum score range of 0 to 48);

Burnout syndrome (BS) was calculated as follows:

- Emotional exhaustion (EE): Total score of 0–11 [Low BS], 12–17 [Moderate BS], ≥18 [High BS].
- Depersonalization (DP): Total score of 0–5 indicated low BS, 6–9 indicated moderate BS, and a score greater than ≥10 indicated high BS.
- Personal accomplishment (PA): A total score of more than 26 indicated low PA, 22 to 26 indicated moderate PA, and 0 to 21 indicated high PA.

### 2.4. Validity and Reliability of Questionnaire

The questionnaire items were adapted from the English version of MBI [1]. The English version was translated into Arabic by a bilingual translator who was a native Arabic speaker and proficient in both Arabic and English. The translated version was validated for content and meaning by a panel of 6 content experts, and validity was assessed using Aiken’s test. A value higher than 0.90 was achieved for each question. The respondents were given the choice of answering either version (English or Arabic) according to their convenience. The scores were calculated according to the original MBI guidelines. To assess reliability, the questionnaire was distributed to 25 subjects. Cronbach’s α of 0.87 was obtained for EE, 0.85 for DP, and 0.83 for PA for each subscale, with an overall value of 0.85 obtained.

### 2.5. Statistical Analysis

Descriptive summary statistics were obtained for all independent and outcome variables related to burnout. The difference in mean scores was tested using a *t*-test and analysis of variance (ANOVA), followed by Tukey’s Post Hoc analysis for inter-variable comparisons. Differences in proportions were tested using the Kruskal–Wallis H test, followed by the Mann–Whitney U test for intergroup comparisons and the Chi-Square test. The Kolmogorov–Smirnov test was used to check the normal distribution of data. Multivariate logistic regression analysis accounting for multicollinearity was carried out for the independent variables (age, gender, marital status, type of participants (students vs. interns/practitioners), work experience (for dental practitioners), number of working hour per week, number of working days per week, and area of work for dental practitioners), and outcome variable of burnout syndrome was taken as dichotomous (present/absent). The presence of burnout syndrome was indicated by a high score of EE (≥18) and DP (≥10), and PA (>26). Variables with a significance level *p* < 0.05 in bivariate analysis were included for regression analysis. The analysis was performed using the Statistical Package for Social Science (SPSS) version 24 (IBM Corp., in Armonk, NY, USA). All statistical tests were two-sided, and the significance level was *p* < 0.05.

## 3. Result

A total of 302 participants (female = 25, male = 277) were included. The mean age of study participants was 30.2 ± 4.1 years. Table 1 shows the prevalence of burnout syndrome among the types of study participants. A total of 66.2% of participants presented with high emotional exhaustion, 48.7% with high depersonalization, and 38.7% with low personal achievement. A total of 84.3% of dental students presented with high EE compared to interns (61.5%) and practitioners (58.9%) (*p* = 0.031). Similarly, 67.5% of dental students presented with high DP, compared to interns (25.6%) and practitioners (45%) (*p* = 0.023). Additionally, 91.6% of dental students presented with low PA, compared to interns (48.7%) and practitioners (54.4%) (*p* = 0.001).

Table 2 shows the mean burnout score among study participants based on the sociodemographic details. The mean emotional exhaustion (2.9 ± 17) and depersonalization score (2.3 ± 1.3) were high among participants of 25–30 years of age. The mean personal achievement score (4.1 ± 2.9, *p* = 0.042) was high among the participants above 40 years of age. The mean emotional exhaustion and depersonalization scores were high among female participants. The students presented with high mean emotional exhaustion and depersonalization scores.

Table 3 shows the mean burnout score based on work experience. The mean emotional exhaustion (3.2 ± 2.1) and depersonalization score (3.4 ± 2.6) were high among participants who worked more than 40 h per week (*p* = 0.001). The mean emotional exhaustion (3.1 ± 1.9) and depersonalization score (3.3 ± 2.1) were high among participants who worked more than 5 days per week (*p* = 0.001). The mean personal achievement score (4.1 ± 2.3) was high among participants who worked less than 5 days a week.

Table 4 shows a multivariate logistic regression model of factors associated with the burnout syndrome. Burnout level was 1.78 times (CI = 1.52–3.53, *p* = 0.032) higher among female participants than males. Burnout level was 1.53 times (CI = 1.31–3.17, *p* = 0.043) higher among student participants than practitioners. Burnout level was 2.72 times (CI = 1.87–3.86, *p* = 0.021) higher among participants who worked more than 40 h per week than those who worked ≤40 h per week. Burnout level was 2.41 times (CI = 1.72–3.79, *p* = 0.023) higher among participants who worked more than 5 days per week than those who worked ≤5 days per week.

## 4. Discussion

The prevalence of burnout has significantly increased among healthcare professionals, including dentists, since the onset of the COVID-19 pandemic [2,19]. Dental professionals are susceptible to burnout, a complex phenomenon influenced by various factors, including patient-related stressors, occupational demands, individual characteristics, and external pressures [20,21]. The present study aimed to assess the prevalence of burnout and identify associated factors among dental students, interns, and practitioners in Saudi Arabia. The overall response rate in this study was 90%, which is notably higher than that reported in previous research, ranging from 48% [22] to 87.5% [23]. This increased participation may be attributable to the personal interviews, which likely enhanced respondent engagement.

Burnout syndrome is characterized by emotional exhaustion [1]. Prior research has indicated that emotional exhaustion among healthcare workers can vary significantly, with reported rates ranging from 38% to 98% [24,25,26]. The current investigation found a rate of emotional exhaustion of 66.2%, consistent with studies conducted in Egypt and Spain, where rates of 62% and 61.3%, respectively, were documented [27,28].

The results of the current study indicate a substantial prevalence of burnout syndrome among dental students, exceeding that of dental interns and practitioners. Burnout level was 1.53 times higher among student participants compared to practitioners. This finding is consistent with the work of Abdul NS et al. [10], who documented a high burnout rate (92.8%) within the dental student population. The rigorous academic demands with the stress of passing exams, coupled with the challenges of clinical training, peer pressure and competition, limited socioeconomic power, and distance from family and home, may exacerbate stress and negative emotions, thereby increasing the risk of burnout among dental students [29,30,31].

Among the sociodemographic factors, age emerged as a significant predictor of burnout. Participants aged 25–30 years reported a 1.52 times higher level of burnout. This finding is consistent with the existing literature, which indicates that younger individuals, particularly those in early career stages, may be more susceptible to burnout due to increased work demands, role ambiguity, and work–life imbalance [31,32,33,34].

Burnout is a significant occupational hazard among dental professionals, and gender appears to play a role in its prevalence and manifestation [1,35,36,37]. While some studies have shown no significant gender differences in burnout among dental students [35], others have reported higher levels of emotional exhaustion and lower levels of personal accomplishment among female dental students [36]. In line with Brake et al. [38] and Oshima et al. [39], the present study demonstrated significant gender disparities in burnout, with female participants reporting 1.78 times higher levels than their male counterparts. Cultural barriers Saudi women face may be considered risk factors that might add to their stress levels and result in an increase in burnout syndrome among women [40].

Several studies have highlighted the role of working hours as a major contributor to burnout in these groups [1,35,36,41,42,43,44]. In line with previous research [41], the present study demonstrated a significant association between increased working hours and elevated burnout. Participants with a workweek exceeding 40 h and a work schedule of more than 5 days per week reported significantly higher burnout scores. Burnout was 2.72 times and 2.41 times higher among participants who worked more than 40 h per week and more than 5 days per week, respectively.

Limitations: While this study provides valuable insights into burnout among dental professionals in Saudi Arabia, it is important to acknowledge its limitations. The cross-sectional design restricts our ability to establish causal relationships, and the self-reported nature of the data may introduce biases. Lack of stratification of study participants resulted in a skewed number of female participants and interns, which might have affected the validity of group comparison. Due to the small proportion of female participants, gender comparisons should be interpreted with caution. Additionally, we did not collect information about whether respondents had children, which could have indirectly influenced burnout levels. Data on nationality (Saudi vs. non-Saudi) were collected but not analyzed due to limited non-Saudi representation.

Moving forward, future research should address these gaps through longitudinal studies to better understand the long-term consequences of burnout on professionals’ well-being, job satisfaction, and patient care. Qualitative approaches could further illuminate the lived experiences of dental professionals and identify specific challenges they face. Future studies should prioritize stratified sampling to explore gender-specific burnout experiences, particularly among Saudi women facing cultural stressors. Additionally, future studies should consider culturally adapting items to align with Saudi norms, such as rephrasing Likert-scale anchors or examples to reflect local workplace dynamics. Finally, future research should examine nationality-based differences to provide a more comprehensive understanding of burnout across diverse groups within the dental profession.

## 5. Conclusions

The present study’s results demonstrated significantly higher prevalence of burnout syndrome among dental students compared to interns and practitioners. Younger age and female gender were significantly associated with increased burnout. A strong correlation was observed between extended weekly working hours and burnout syndrome. To mitigate these effects, implementation of targeted interventions is essential, including the following:

1. Stress management programs;
2. Work–life balance initiatives;
3. Supportive workplace environments.

By addressing these modifiable risk factors through evidence-based interventions, healthcare institutions can potentially reduce burnout prevalence and enhance the professional well-being of dental practitioners in Saudi Arabia.

### Key Statistical Findings

The students presented with high mean emotional exhaustion and depersonalization scores. Burnout level was 1.53 times (CI = 1.31–3.17, *p* = 0.043) higher among student participants than practitioners. The mean emotional exhaustion and depersonalization scores were high among female participants compared to male participants. Burnout level was 1.78 times (CI = 1.52–3.53, *p* = 0.032) higher among female participants than male participants. The mean emotional exhaustion and depersonalization score was high among participants who worked more than 40 h and more than 5 days per week. Burnout level was 2.72 times (CI = 1.87–3.86, *p* = 0.021) and 2.41 times (CI = 1.72–3.79, *p* = 0.023) higher among participants who worked more than 40 h and more than 5 days per week.

## Figures and Tables

**Table 1:** Distribution of burnout syndrome as per the type of study participants.

Participant Type	EE			DP			PA			
	Low	Moderate	High	Low	Moderate	High	Low	Moderate	High	Chi-Square *p* Value
Students (*n* = 83)	7	6	70	21	6	56	76	7	0	0.041
Percentage	8.4	7.2	84.3	25.3	7.2	67.5	91.6	8.4	0	
Interns (*n* = 39)	15	0	24	5	24	10	19	20	0	
Percentage	38.5	0	61.5	12.8	61.5	25.6	48.7	51.3	0	
Practitioners (*n* = 180)	45	29	106	58	41	81	98	52	30	0.032
Percentage	25	16.1	58.9	32.2	22.8	45	54.4	28.9	16.7	
Total (*n* = 302)	67	35	200	84	71	147	117	79	106	
Percentage	22.2	11.6	66.2	27.8	23.5	48.7	38.7	26.2	35.1	
Kruskal–Wallis H test	0.031			0.023			0.001			
Mann–Whitney U test	Students > Practitioners and interns			Students > Practitioners and interns			Students > Practitioners and interns			

EE = Emotional exhaustion. DP = Depersonalization. PA = Personal achievement.

**Table 2:** Mean burnout score among study participants as per the sociodemographic details.

Details of Study Participants	Burnout Category					
	Emotional Exhaustion	Depersonalization	Personal Achievement	ANOVA F Value	Statistical Significance	Tukey’s Post Hoc
Age in years						
25–30 years (*n* = 189)	2.9 (1.7)	2.3 (1.6)	3.9 (2.2)	5.921	0.042 *	25 to 30 yr > 31 to 40 yr
31–40 years (*n* = 92)	2.6 (1.8)	1.9 (1.5)	3.7 (2.1)			
>40 years (*n* = 21)	2.5 (1.1)	2.1 (1.3)	4.1 (2.9)			
Gender						
Male (*n* = 277)	1.8 (0.7)	1.3 (0.8)	3.8 (2.1)	NA	0.03 **	NA
Female (*n* = 25)	1.9 (1.7)	2.3 (1.9) *	3.8 (2.3)			
Marital status						
Married (*n* = 161)	2.6 (1.3)	1.0 (1.1)	3.5 (2.2)	NA	0.031 **	NA
Single (*n* = 141)	2.9 (1.8)	2.4 (1.7)	4.1 (2.8)			
Type of participants						
Students (*n* = 83)	3.3 (1.9)	2.6 (1.7)	3.6 (2.1)	5.963	0.032 *	S > P
Interns (*n* = 39)	2.2 (1.6)	2.1 (1.4)	3.7 (2.1)			
Practitioners (*n* = 180)	2.6 (1.1)	2.0 (1.2)	4.3 (2.9)			

S = Students, P = Practitioners, ANOVA = Analysis of variance, NA = Not applicable, * = *p* value of ANOVA, ** = *p* value of *t*-test.

**Table 3:** The mean burnout score based on work experience.

Work Experience	Burnout Category			Statistical Significance
	Emotional Exhaustion	Depersonalization	Personal Achievement	
Work experience for dental practitioners				*t*-test
Less than 10 years (*n* = 219)	2.8 (1.4)	2.2 (1.3)	3.9 (2.2)	0.121
≥10 years (*n* = 83)	2.6 (1.1)	2.0 (0.7)	3.8 (1.9)	
Number of working hours per week				*t*-test
≤40 (*n* = 268)	2.1 (0.9)	2.2 (0.9)	3.9 (2.5)	0.001
More than 40 (*n* = 34)	3.2 (2.1)	3.4 (2.6)	3.7 (2.2)	
Number of working days per week				*t*-test
≤5 days (*n* = 256)	2.4 (1.1)	2.3 (0.9)	4.1 (2.3)	0.001
More than 5 days (*n* = 46)	3.1 (1.9)	3.3 (2.1)	3.7 (2.2)	
Area of work for dental practitioners				ANOVA
Ministry of Health (*n* = 119)	2.8 (1.7)	2.2 (0.8)	3.4 (2.1)	F value = 3.214, *p* = 0.081
Ministry of Education (*n* = 75)	2.3 (1.1)	1.9 (0.7)	4.2 (2.3)	
Both (*n* = 108)	2.7 (1.4)	2.6 (1.1)	3.8 (2.2)	

**Table 4:** Multivariate logistic regression model of factors associated with burnout syndrome.

Variables	Adjusted R^2^	95% CI	*p* Value	VIF
Age in years				
25 to 30 years	1.52	1.42–2.98	0.045	1.7
≥31 years ^#^	1.00			
Gender				
Male ^#^	1.00			
Female	1.78	1.52–3.53	0.032	1.3
Marital status				
Married	0.87	0.13–1.94	0.083	1.2
Single ^#^	1.00			
Type of participants				
Students	1.53	1.31–3.17	0.043	1.4
Interns	0.85	0.34–1.82	0.091	
Practitioners ^#^	1.00			
Work experience for dental practitioners				
Less than 10 years	0.89	0.35–2.12	0.063	1.3
≥10 years ^#^	1.00			
Number of working hours per week				
≤40 h ^#^	1.00			
More than 40 h	2.72	1.87–3.86	0.021	1.4
Number of working days per week				
≤5 days ^#^	1.00			
More than 5 days	2.41	1.72–3.79	0.023	1.8
Area of work for dental practitioners				
Ministry of Health ^#^	1.00			
Ministry of Education	0.65	0.11–1.78	0.142	1.4
Both	0.94	0.34–2.03	0.059	1.5

^#^ = Reference value, CI = Confidence interval, VIF = Variance inflation factor.

## Data Availability

The original contributions presented in this study are included in the article. Further inquiries can be directed to the corresponding author.
